# IC2 participates in the cooperative activation of outer arm dynein densely attached to microtubules

**DOI:** 10.1247/csf.23044

**Published:** 2023-07-27

**Authors:** Yusuke Kondo, Tomoka Ogawa, Emiri Kanno, Masafumi Hirono, Takako Kato-Minoura, Ritsu Kamiya, Toshiki Yagi

**Affiliations:** 1 Department of Life and Environmental Sciences, Faculty of Bioresource Sciences, Prefectural University of Hiroshima, Shobara, Hiroshima 727-0023, Japan; 2 Department of Biological Sciences, Chuo University, Kasuga, Tokyo 112-8551, Japan; 3 Department of Frontier Bioscience, Hosei University, Koganei, Tokyo 184-8584, Japan

**Keywords:** cilia, axoneme, dynein heavy chain, cooperativity

## Abstract

Ciliary outer-arm dynein (OAD) consists of heavy chains (HCs), intermediate chains (ICs), and light chains (LCs), of which HCs are the motor proteins that produce force. Studies using the green alga *Chlamydomonas* have revealed that ICs and LCs form a complex (IC/LC tower) at the base of the OAD tail and play a crucial role in anchoring OAD to specific sites on the microtubule. In this study, we isolated a novel slow-swimming *Chlamydomonas* mutant deficient in the IC2 protein. This mutation, E279K, is in the third of the seven WD repeat domains. No apparent abnormality was observed in electron microscope observations of axonemes or in SDS-PAGE analyses of dynein subunits. To explore the reason for the lowered motility in this mutant, *in vitro* microtubule sliding experiments were performed, which revealed that the motor activity of the mutant OAD was lowered. In particular, a large difference was observed between wild type (WT) and the mutant in the microtubule sliding velocity in microtubule bundles formed with the addition of OAD: ~35.3 μm/sec (WT) and ~4.3 μm/sec (mutant). From this and other results, we propose that IC2 in an OAD interacts with the β HC of the adjacent OAD, and that an OAD-OAD interaction is important for efficient beating of cilia and flagella.

## Introduction

Cilia and flagella (interchangeable terms) are motile organelles of eukaryotic cells. They produce rhythmic bending waves to produce water flow, allowing cells to move themselves or transport extracellular substances. Dysfunction of these organelles leads to human conditions known as primary ciliary dyskinesia (PCD) or immotile cilia syndrome (Afzelius, 2004). PCD patients exhibit various types of symptoms, such as infertility, bronchiectasis, or abnormal left-right asymmetry of internal organs (Reiter and Leroux, 2017; Sironen *et al.*, 2020). Cilia and flagella of most organisms share a common cytoskeletal structure called the axoneme, consisting of nine doublet microtubules surrounding a pair of singlet microtubules. The A-subfiber of each doublet microtubule attaches two types of dynein assemblies, inner-arm dynein (IAD) and outer-arm dynein (OAD), both of which are composed of multiple subunits including force generating heavy chains (HCs). Mutants lacking IAD display slow swimming due to a reduction in the bend amplitude, while mutants lacking OAD swim slowly due to a reduction in beat frequency (Brokaw and Kamiya, 1987).

*Chlamydomonas* OAD comprises three (α, β and γ) HCs, two intermediate chains (IC1 and IC2), and more than ten light chains (LC1–LC10 and homologues of some LCs) (King *et al.*, 2023). Each HC consists of a head region, which corresponds to the C-terminal two-thirds of the sequence, and a tail region, which corresponds to the N-terminal one-third. The head region possesses ATPase, microtubule-binding, and motor activities, while the tail region is responsible for ATP-independent binding to the doublet microtubule. The two ICs and various LCs form a complex (IC/LC complex; also called the IC/LC tower for its structure) and bind to the OAD tail region (King, 2012; Rao *et al.*, 2021; Walton *et al.*, 2021; Kubo *et al.*, 2021). Lack of ICs leads to a complete loss of OAD (Kamiya and Yagi, 2014; King *et al.*, 2023), suggesting that the IC/LC complex is essential for anchoring OAD structure to specific sites on doublet microtubules. The ICs and LCs interact not only with each other in the same OAD assembly, but also with other axonemal proteins including neighboring IAD or OAD (Nicastro *et al.*, 2006; Ishikawa *et al.*, 2007; Oda *et al.*, 2013; Rao *et al.*, 2021; Walton *et al.*, 2021; Kubo *et al.*, 2021; Walton *et al.*, 2023). An OAD complex is likely to associate with an adjacent OAD complex on the outer doublet microtubule (Haimo *et al.*, 1979; Haimo and Fenton, 1984), possibly based on multiple interaction sites including LCs and ICs (Rao *et al.*, 2021; Walton *et al.*, 2021; Kubo *et al.*, 2021; Zimmermann *et al.*, 2023). Such inter-OAD interaction has been considered important for the activation of dynein motility (Aoyama and Kamiya, 2010).

In this study, we isolated a new type of mutant exhibiting a moderately reduced swimming speed, i.e., a speed between wild type (WT) and *oda* mutants lacking OAD. The new mutant was found to carry a point mutation within one of the WD repeat domains in IC2. This is the first time that a point mutation in IC2 has been shown to reduce the overall motile activity of axoneme. The axonemes isolated from this mutant underwent sliding disintegration less frequently than WT axonemes when ATP was added in the presence of protease. Interestingly, unlike wild-type OAD, the mutant OAD did not show a large increase in the velocity of microtubule sliding observed when OADs are densely packed on the microtubule. These observations suggest that this novel IC2 mutation affects the OAD and axoneme activities mainly through its impaired interaction with adjacent OAD molecules. It is plausible that IC2 is involved in the interaction between OAD molecules and that such interaction enhances the axonemal motility.

## Materials and Methods

### Strains and culture conditions

*Chlamydomonas reinhardltii* strains used were WT (CC-124 and CC-125), mutants lacking OAD (*oda1*, *oda2*, *oda6*, *oda11*, *oda4-S7*, *oda2-t*) (Kamiya, 1988; Sakakibara *et al.*, 1991, 1993; Liu *et al.*, 2008), an allele of *oda6*, *oda6-r88* (Mitchell and Kang, 1993), and mutants lacking IAD (*ida1*, *ida4*, *ida5*, and *ida9*) (Kamiya *et al.*, 1991; Kato *et al.*, 1993; Kato-Minoura *et al.*, 1997; Yagi *et al.*, 2005). Double mutants between two dynein-deficient mutants were constructed by a standard method (Harris, 2009). Cells were cultured in Tris-Acetate-Phosphate medium with aeration under a 12 h/12 h light/dark cycle at 25°C (Gorman and Levine, 1965).

### Mutant isolation

Non-motile mutants were produced by UV-mutagenesis of the IAD mutant *ida5*. The mutants were crossed with WT to remove the *ida5* mutation. The recovered motile mutants were further backcrossed with WT twice, and their motility and axonemal proteins were analyzed.

### Analysis of the mutation

Mutation was mapped using an ampliﬁed-fragment-length polymorphism (AFLP) analysis based on genetic crossing between polymorphic strains (Kathir *et al.*, 2003). DNA fragments of several candidate genes in the mapped region were amplified by PCR using KOD-FX (KFX-101, TOYOBO). The amplified DNA fragments were analyzed with a DNA sequencer, ABI PRISM 3100 (Thermo Fisher Scientific).

### Transformation of mutant cells

The genomic DNA covering the *IC2* gene was cloned into pBluescript II vector using InFusion HD (Clontech Lab.). The DNA was amplified from WT genome DNA by PCR, using PrimeSTAR GXL (R050A, Takara). Primers used were 5'-GAGATGAACAGGTATTGGTGACGAG-3' and 5'-GACTTGATACCCTTCCTGCAGTTC-3'. The plasmid was digested with EcoRI. The resultant fragment containing the WT *IC2* gene was sub-cloned into the EcoRI site of the pSI103 plasmid harboring paromomycin-resistant gene (Sizova *et al.*, 2001). The plasmid containing the genomic *IC2* gene was then transformed into the double mutant *ida4; oda6* or *ida4; 214* (*oda6E279K*) by electroporation (Yamano *et al.*, 2013). Colonies were selected on agar plates with paromomycin. Positive clones containing the *IC2* gene were selected by PCR analysis.

### Motility assessment

Cells were observed at 25°C under red light illumination using a dark-field microscope (BX50, Olympus). Images of cell movements were taken with a CCD video camera (Model CS230, Olympus). The images were digitized using a video A/D converter (IC Imaging Control 3.0, The Imaging Source). The swimming velocity of cells was obtained using ImageJ software (https://imagej.nih.gov/ij/index.html) with the plug-in program ‘MTrack2’. Flagellar beat frequency was measured from the vibration in the images of swimming cells using Fast Fourier Transform (Kamiya, 2000). The average of median beat frequencies for a given strain was obtained from three independent measurements using different cell cultures.

### Preparation of flagellar axonemes

Flagellar axonemes were prepared by the dibucaine method of Witman (1986). Briefly, flagella were detached from cells with dibucaine-HCl (FUJIFILM Wako Pure Chemical, Tokyo), and demembranated with 1% Nonidet P-40 in HMDEK buffer (30 mM HEPES-NaOH (pH 7.4), 5 mM MgSO_4_, 1 mM DTT, 1 mM EGTA, and 50 mM CH_3_COOK).

### Electron microscopy

Axoneme samples were fixed with 2% glutaraldehyde in the presence of 2% tannic acid, post-fixed with 1% OsO_4_, dehydrated through a series of ethanol solutions, and embedded in Epon 812. Gray/silver sections approximately 50-nm-thick were cut, double-stained with uranyl acetate and lead citrate. Microtubule bundles formed with OAD were observed by negative stain EM. The microtubule bundles were fixed with 2% glutaraldehyde and stained with 1% uranyl-acetate on carbon-coated grids. All samples were observed with a JEM-1010 microscope (JEOL Co., Tokyo).

### SDS-PAGE and immunoblot analyses

For the analysis of dynein HCs, axoneme or fractionated samples was subjected to SDS-PAGE with a 4% acrylamide and 4 M urea gel (King *et al.*, 1986). Dynein LCs (8–25 kDa) in mutant axonemes was analyzed by SDS-PAGE using a 5–15% acrylamide gradient gel. The gels were stained with either Coomassie Brilliant Blue or silver. For immunoblot analysis, proteins separated on the SDS-PAGE gel were transferred to PVDF membranes using Semi-dry electro-blotter (Transblot SD, BioRad). The membrane was processed with primary antibodies (polyclonal antibody against IC2 protein) and secondary antibodies conjugated with horseradish peroxidase (NA934; GE Healthcare). Immunologically positive proteins were detected using a chemiluminescence reagent (Chemilumi One Super, Nakali Tesque, Kyoto), and a chemiluminescence imager (WSE-6200, ATTO, Tokyo).

### Detergent-extracted cells (cell models)

Cell model experiments were performed by the standard method (Kamiya and Witman, 1984) with minor modifications. In brief, cells were washed twice with HES buffer (10 mM HEPES-NaOH (pH 7.4), 2 mM EGTA, and 4% sucrose) and demembranated with 0.5% Nonidet P-40 in HMDEK. Demembranated cells were diluted into HMDEKP (HMDEK plus 1% polyethylene glycol). The motility of cell models was reactivated in the presence of 0.05–1.0 mM ATP. An ATP regeneration system consisting of 70 U/ml creatine kinase (CK-RO, Sigma) and 5 mM phosphocreatine (300-50521, FUJIFILM Wako Pure Chemical) was used to maintain the ATP concentration.

### Sliding disintegration of axoneme

Sliding disintegration of axonemes was induced and recorded following the method of Okagaki and Kamiya (1986). Axonemes were fragmented by sonication and induced to disintegrate into doublet microtubules by addition of 0.5 μg/ml trypsin in HMDEKP solution containing 1 mM ATP or 1 mM ATP + 1 mM ADP. Microtubule sliding was observed with a dark-field microscope equipped with a 50× oil-immersion objective lens and a 100-W mercury lamp (U-RFL-T, Olympus). Axonemes that were disintegrated into more than two groups of microtubules were counted as ‘disintegrated’ axonemes.

### *In vitro* motility assay

Motor activity of OAD was analyzed by *in vitro* motility assays (Kagami and Kamiya, 1992). Axonemal dyneins were extracted from flagellar axonemes in HMDEK with 0.6 M NaCl for 10 min on ice (Sakakibara and Kamiya, 1989). From this crude dynein extract, OADs were purified by ion-exchange column chromatography (Kagami and Kamiya, 1992). OAD was adsorbed on the glass surface of a flow chamber made of a glass slide, a coverslip, and a pair of spacer strips. Microtubules assembled from porcine brain tubulin and stabilized with Taxol were perfused into the chamber, and microtubules bound to the glass-adsorbed dynein were observed with a dark-field microscope. Microtubule gliding on the glass surface was initiated by the addition of 1 mM ATP + 1 mM ADP. The gliding movements were video-recorded and the microtubule gliding speed was measured using the ImageJ software.

### Sliding in microtubule bundles

Motor activity of OAD was also analyzed by measuring the velocity of microtubules extrusion from microtubule bundles. Bundles with densely attached OADs were produced by the method of Aoyama and Kamiya (2010). To induce OAD-microtubule binding by the ATP-insensitive site (i.e., by the OAD tail), crude axonemal extracts containing OAD were added to polymerized and Taxol-stabilized porcine brain microtubules, which had been adsorbed on the glass surface in the presence of ATP. After washing out ATP and introducing additional microtubules, microtubule bundles were formed. In such bundles, microtubule extrusion was initiated by perfusing the observation chamber with 1 mM ATP solution. The sliding movements were video-recorded.

## Results

### Novel mutant apparently deficient in OAD function

**Fig. 1:**
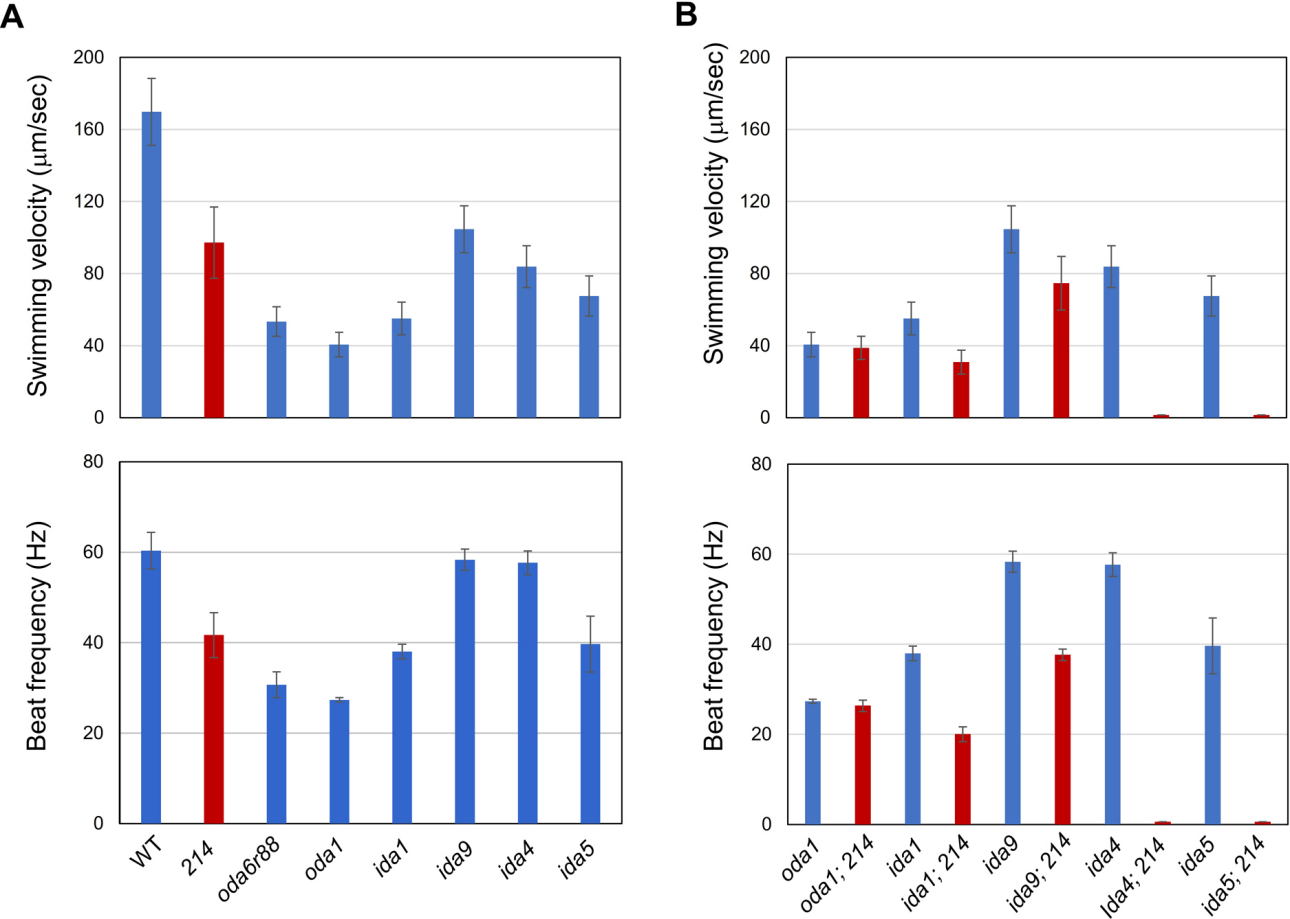
Motility of the novel mutant *214* compared with other strains (A) Swimming velocity (upper figure) and beat frequency (lower figure) in *214* and various dynein-deficient mutants. (B) Swimming velocity and beat frequency in various dynein-deficient mutants with and without the *214* mutation. Double mutants between *214* and IAD mutants (*ida4*, *ida5*) were nonmotile. In contrast, double mutant between *214* and the mutant *oda1* lacking OAD swam at almost the same velocity as that of *oda1*. Average ± SEM of swimming velocity was measured in >30 cells for each strain. Average ± SEM of beat frequency in a population cells (>30 cells) was estimated from the beat frequency distribution obtained from Fast Fourier Transform analyses of cell body vibration.

To explore the regulation and coordination in OAD, we tried to isolate novel mutants that display phenotypes typical of OAD deficiency. For this purpose, we first UV-mutagenized the mutant *ida5* lacking several IAD species (Kato *et al.*, 1993), and screened for non-motile cells. This is because many OAD-deficient mutants were found to become non-motile when combined with such an IAD-deficient mutation (Kamiya *et al.*, 1991). The isolated nonmotile cells were crossed with WT, and a slower-swimming mutant (tentatively named *214*) was obtained among the progenies. Its swimming velocity (~95 μm/s) and beat frequency (~40 Hz) were about 60% and 65% of those of WT, respectively (Fig. 1A). Notably, the beat frequency was higher than that of the mutant *oda1*, which lacks the entire outer-arm dynein (~25 Hz) (Kamiya, 1988; Fig. 1A). It became non-motile when combined with the *ida4* mutation (lacking IADs a, c, and d), and displayed slower swimming when combined with the *ida1* or *ida9* mutations (lacking IAD f/I1 or IAD c) (Fig. 1B). In contrast, the double mutant of *214* and *oda1* showed swimming speed and beat frequency almost the same as those of *oda1* (Fig. 1B), indicating that the motility deficiency in *214* is caused by a defect in OAD rather than IAD.

### Lack of noticeable abnormality in the mutant OAD structure

**Fig. 2:**
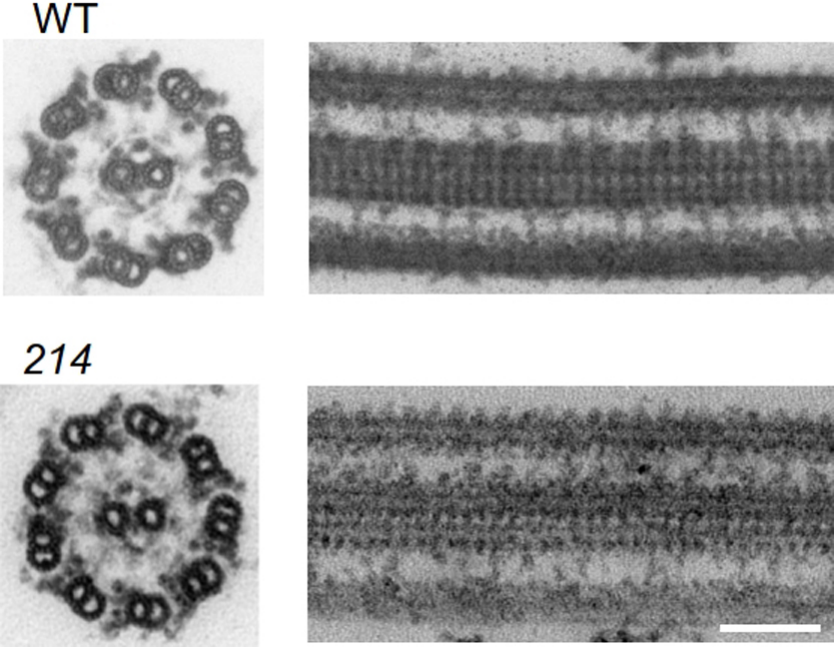
Axoneme images in WT and the mutant *214* Axial and longitudinal sections of axonemes of WT and *214* (*oda6E279K*). Scale Bar: 100 nm.

To examine whether the mutant *214* has an alteration in the OAD structure, we first analyzed its axonemes by electron microscopy. However, the mutant *214* exhibited normal OAD images in axonemal cross sections (Fig. 2). We next examined its axonemal proteins by SDS-PAGE, but the results showed no difference between *214* and WT in the band intensities of the OAD heavy chains (α, β, and γ HCs) (Fig. S1). Further, we also examined the relative affinities in microtubule binding of the mutant and wild-type OADs by a co-pelleting assay, using a mixture of microtubules and axonemal crude extracts. However, the amount of the mutant OAD binding to microtubules was found to be almost the same as that of the wild-type OAD (Fig. S2), indicating that the *214* mutation dose not significantly influence the affinity of OAD binding to microtubules. Finally, we examined the axonemal amounts of ICs and LCs. This is because the mutant *oda6r88*, which has a 23 amino-acid substitution in the N-terminal region of its IC2 (Mitchell and Kang, 1993), was reported to display *oda*-like slow motility possibly due to the absence of LC2, LC6, and LC9 (Dibella *et al.*, 2005). Again, however, SDS-PAGE and immunoblot analyses detected no reduction in IC1, IC2, or LC1–LC6 in *214* (Fig. S3). Thus, the *214* mutation appears to influence OAD function without any noticeable change in these subunits.

### Identification of a mutation in the gene of IC2

Amplified-fragment-length polymorphism (AFLP) analysis (Kathir *et al.*, 2003) mapped the *214* mutation in a 2.0–2.5 Mbp region on chromosome 12 (Fig. S4A). This region contained six genes known to encode flagellar components, including the gene of IC2 located at the *ODA6* locus (Fig. S4A; Mitchell and Kang, 1991). Sequence analyses of the mutant genome DNA revealed a point mutation in the IC2 gene at the 1237th nucleotide from the start codon ATG, which results in a Glu to Lys change at the 279th residue (Fig. S4B). This mutation is positioned in the third of the seven WD repeat domains in IC2 (Fig. S4C). The amino acid Glu at this position is conserved among many organisms except zebrafish, in which it is substituted by Asp (Fig. S5). It is interesting to note that the human IC2 mutation R247Q, located at the boundary between the second and the third WD domains, was previously identified as a causative mutation in a PCD patient who had a reduced number of OADs in trachea cilia (Al-Mutairi *et al.*, 2022).

**Fig. 3:**
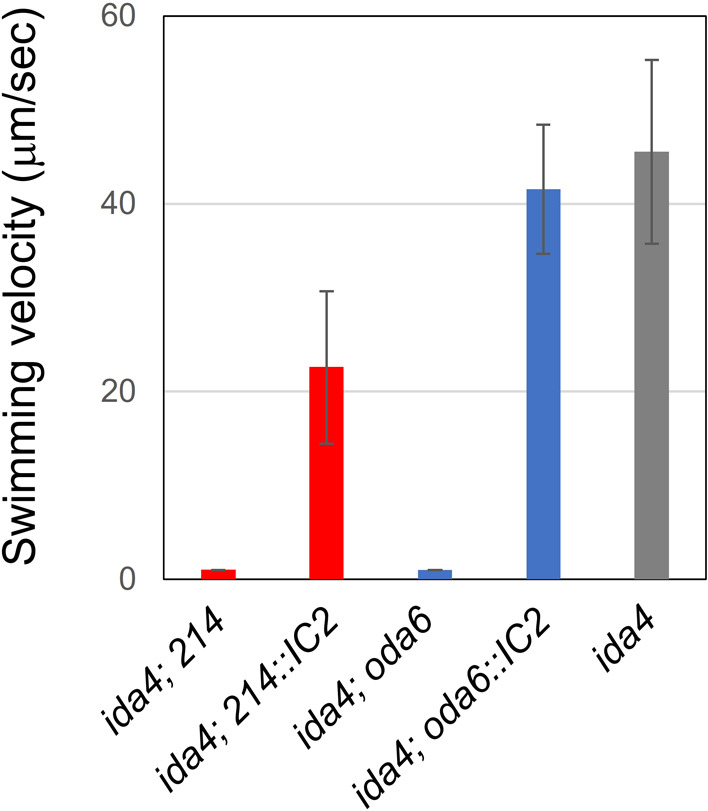
Motility of the double mutant *ida4; 214* transformed with the wild-type *IC2* gene The non-motile mutant *ida4; 214* (*oda6 E279K*) was transformed with the wild-type genomic *IC2* gene. The transformant, *ida4; 214::IC2*, displayed swimming (Movie S1 and S2).

To find out whether the identified point mutation is responsible for the reduced motility of *214*, we carried out transformation of *214* and *oda6* with the wild-type *IC2* gene. In this experiment, we used double mutants of respective strains with *ida4*, an IAD-deficient mutant, because the double mutants are non-motile and rescue of the OAD function is easily detected as a recovery of motility. When the non-motile double mutants *ida4; 214* and *ida4; oda6* were transformed with the wild-type genomic *IC2* gene, they recovered motility (Fig. 3, Movie S1 and S2), indicating that the phenotype of *214* in fact results from the mutation detected in the *IC2* gene. Therefore, we renamed the mutant as *oda6E279K*. The swimming speed of the *214* (*oda6E279K*) transformant was slower than that of the *oda6* transformant. This is probably because the former transformant expressed two types of OADs having either a mutant or a wild-type IC2. Using a BCCP (biotin carboxyl carrier protein)-tagged *IC2* gene construct, we estimated that the *214* transformant expressed these OADs in similar amounts (Fig. S6).

### *In vitro* motility assays of the wild-type and mutant OADs

**Fig. 4:**
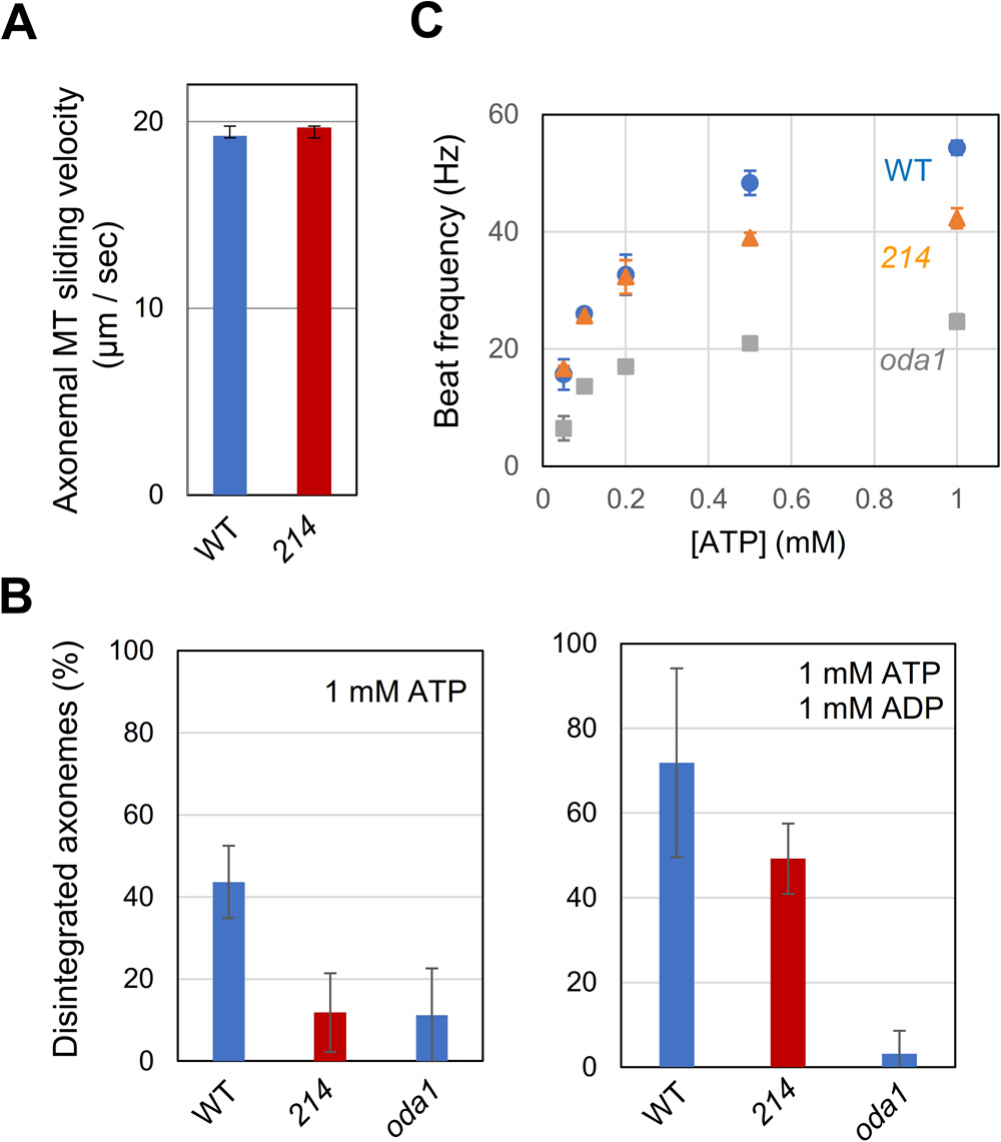
Comparison of axonemal sliding disintegration and beat frequencies ATP-induced sliding speed and sliding frequency in the protease-treated axonemes were measured in WT, the mutant *214* (*oda6E279K*) *and oda1*. (A) Microtubule sliding velocity in *214* (*oda6E279K*) axonemes was almost the same as that in WT axonemes. (B) Frequency of sliding disintegration in proteolyzed axonemes in the presence of 1 mM ATP (Left) and 1 mM ATP and 1 mM ADP (Right). Microtubule sliding activity in *214* axonemes increased in the simultaneous presence of ATP and ADP. (C) ATP-dependence of the axonemal beat frequencies in reactivated cell models of WT, *214* and *oda1*. Beat frequency of *214* (*oda6E279K*) is lower by ~10 Hz than that of WT at 0.5 mM or 1 mM ATP, but it is almost the same as that of WT at 0.05–0.2 mM ATP.

To compare OAD motor activities between *oda6E279K* and WT, microtubule sliding in protease-treated axonemes was investigated. The sliding speed of the mutant was almost the same as that of WT at 1 mM ATP (Fig. 4A), but the number of disintegrated axonemes was significantly lower in the mutant (Fig. 4B). The number, however, increased by the addition of 1 mM ADP, which is known to enhance the activity of axonemal dyneins (Omoto *et al.*, 1996; Yagi and Kamiya, 1995). The flagellar beat frequency in detergent-extracted and reactivated *oda6E279K* cells was significantly lower than that of WT at physiological concentrations of ATP. However, the difference in beat frequency disappeared at low ATP concentrations (Fig. 4C), suggesting that the OAD activity of the mutant is possibly activated at low ATP. Such an effect of ADP or low ATP concentration was not observed in the OAD-lacking axonemes of *oda1* (Fig. 4B and 4C). Thus, it is likely that the addition of ADP or a decrease in the ATP concentration activated microtubule sliding through OAD, and that this is achieved by a change in the nucleotide species bound at the regulatory nucleotide binding site in three AAA domains, AAA2-AAA4 (Yagi, 2000; Shiroguchi and Toyoshima, 2001; Inoue and Shingyoji, 2007). These results suggest that the OAD motor activity of the mutant was suppressed in the presence of a high concentration of ATP, but that the suppression was removed by the addition of ADP or a decrease in the ATP concentration.

**Fig. 5:**
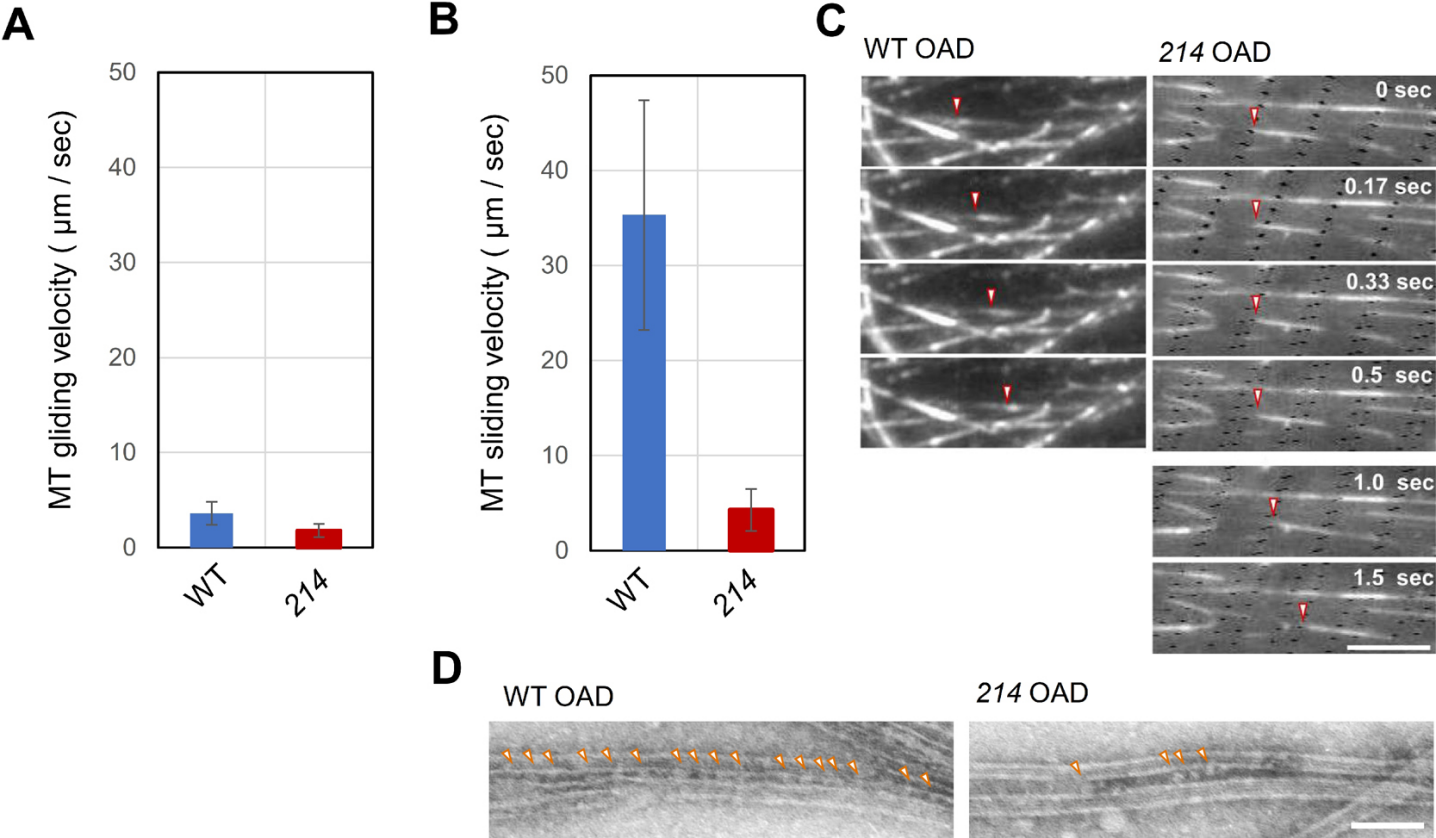
Microtubule movement on the glass surface and in microtubule bundles (A) Velocity of microtubule gliding produced by WT and mutant OADs. OAD αβ complex purified by Mono-Q ion exchange chromatography was randomly attached on to the glass surface and microtubule gliding was induced by addition of 1 mM ATP and 1 mM ADP. The gliding speed with the mutant OAD was approximately 50% slower than with the wild-type OAD. (B) Microtubule sliding speed during ATP-induced dissociation of microtubule bundles formed with wild-type and mutant OADs (αβγ complex). (C) Dark-field images of microtubule sliding in bundles. Arrows indicate the end of a moving microtubule segment. Scale bar: 10 μm. (D) Negative-stain EM images of the microtubule bundles formed with wild-type and mutant dyneins. OAD molecules are periodically and densely aligned between microtubules, as observed by Aoyama and Kamiya (2010). However, the alignment of the mutant OAD is less regular and more sparce than that of the wild-type OAD. OAD molecules were indicated by orange arrowheads. Scale bar: 100 nm.

To compare the OAD activity more directly between *oda6E279K* and WT, an *in vitro* assay was carried out to measure the speed of microtubule gliding driven by isolated OAD (Kagami and Kamiya, 1992; Sakakibara and Nakayama, 1998). A glass slide was coated with the OAD αβ complex purified by ion exchange chromatography, and microtubule gliding was video-recorded under a dark-field microscope. In the presence of 1 mM ATP and 1 mM ADP, the microtubule gliding speed with mutant OAD (1.8 ± 0.7 μm/sec) was 50% slower than that with wild-type OAD (3.6 ± 1.2 μm/sec) (Fig. 5A), suggesting that OAD motor activity is lowered by the IC2 point mutation.

### Lack of inter-molecular activation in mutant OAD

OAD molecules are known to attach with each other when aligned on microtubules in the axoneme, and this inter-molecular association has been suggested to activate their function (Aoyama and Kamiya, 2010; Rao *et al.*, 2021; Walton *et al.*, 2021; Kubo *et al.*, 2021). To examine whether such activation is affected in the mutant OAD, we investigated ATP-induced sliding in bundles of microtubules formed with OADs. Crude axonemal extracts containing OAD were added to microtubules adsorbed on the glass surface in the presence of ATP so as to induce OAD binding to the microtubules by the ATP-insensitive site (Aoyama and Kamiya, 2010). Removal of ATP and further addition of microtubules caused formation of microtubule bundles. In such bundles, OADs are periodically arranged between microtubules (Fig. 5D; Aoyama and Kamiya, 2010). Perfusion with 1 mM ATP induced rapid microtubule sliding, with a speed significantly faster than the gliding speed of microtubules on a glass-surface (Fig. 5B). Interestingly, the microtubule sliding speed in the microtubule bundles formed with the mutant OAD, 4.3 ± 2.2 μm/sec, was about eight times slower than that in the bundles formed with the wild-type OAD, 35.3 ± 12.1 μm/sec (Fig. 5B and 5C).

The arrangement of the OAD molecules in microtubule bundles was examined by negative-stain EM. Both wild-type and mutant OADs showed periodical arrangement on the microtubules. However, the number of continuously aligned OAD molecules between microtubules was somewhat lower in the mutant OAD than in wild-type OAD (Fig. 5D). Although quantitative comparison of arrangement between the two species of OAD is a subject of future studies, this preliminary result seems to suggest that the interaction between the mutant OAD molecules, or between the mutant OAD and microtubules is weaker or more irregular than that in wild-type OAD. In any case, it is likely that the E279K mutation has resulted in a weakening of the inter-OAD interaction as well as a significant reduction in the OAD motor activity.

### Which OAD HC function is most affected by the IC2 mutation?

**Fig. 6:**
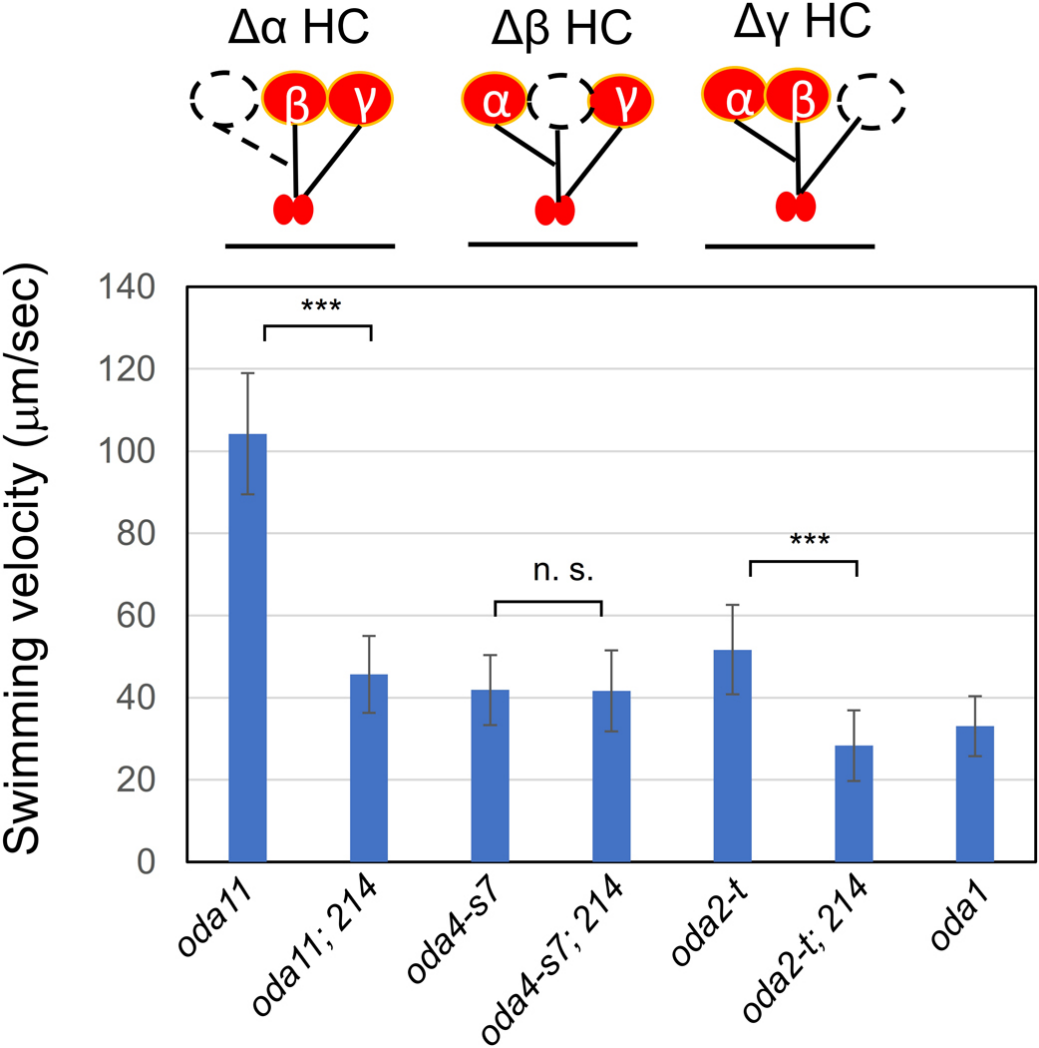
Effect of the E279K mutation on the swimming speed of mutants lacking a particular OAD HC Swimming velocities in the double mutants of *214* (*oda6E279K*) with one of the following strains were measured in >30 cells for each: *oda11* lacking the αHC, *oda4-s7* lacking the motor domain of the β HC, and *oda2-t* lacking the motor domain of the γ HC. While two species of double mutants, *214; oda11* and *214; oda2-t* swam more slowly than the respective single mutants, the double mutant *214; oda4-S7* swam at almost the same velocity as *oda4-S7*. Statistical significance was analyzed by the Student’s t-test. ***, P<0.001; n.s., not significant.

Which of the α, β, and γ HCs of OAD is functionally most affected by the IC2 mutation? To explore this question, we genetically combined the *oda6E279K* mutant with dynein mutants deficient in one of the three HCs and analyzed their phenotypes (Fig. 6). We found that the double mutant with *oda11* (lacking the α HC) *or* with *oda2-t* (lacking the motor domain of γ HC) displayed slower swimming than the respective single mutant, whereas the double mutant with *oda4-S7* (lacking the motor domain of β HC), displayed almost the same swimming velocity as that of the original *oda4-S7* mutant. The result suggests that the IC2 point mutation reduced the OAD motor activity by primarily affecting the interaction with β HC.

## Discussion

### Motor activity of the mutant OAD

Several OAD mutants lacking IC or LC have previously been isolated and characterized (King *et al.*, 2023). Most of the mutants lack the entire OAD or have a greatly reduced number of OAD assemblies and swim more slowly, with a flagellar beat frequency reduced to 30–40% of the WT value (Fig. 1A). The reduced OAD number in these mutants has implied that the IC/LC complex is important for OAD to attach to doublet microtubules, an idea also inferred from the location of the IC/LC complex attached at the base of an OAD molecule (King and Witman, 1990; Walton *et al.*, 2021; Rao *et al.*, 2021; Kubo *et al.*, 2021). In contrast, the mutant *oda6E279K* (*214*) has an apparently complete set of OAD components despite its significantly lower swimming speed than that of WT. This suggests that the mutation has little effect on the targeting of OAD but somehow affects the motor activity of OAD.

The IC2 E279K mutation reduces the OAD motor activity, rather than completely inhibits it. Microtubule sliding in protease-treated axonemes was much less frequently observed in the *oda6E279K* axonemes than in WT axonemes (Fig. 4B). Interestingly, however, the frequency of sliding events increased to the WT level by the addition of ADP. Also, the flagellar beat frequency in detergent-extracted and reactivated *oda6E279K* cells was significantly lower than that of WT at physiological concentrations of ATP, but not at low ATP concentrations (Fig. 4C). The reduced OAD motor activity in the mutant could thus be almost restored to the wild-type level in the presence of ATP and ADP or very low concentration of ATP. These nucleotide conditions have previously been shown to increase OAD and IAD activities in the wild-type and mutant axonemes, possibly through some allosteric effect on dynein HCs, which have three ATP/ADP binding sites in addition to a catalytic site (Omoto *et al.*, 1996; Yagi and Kamiya, 1995). As in previous studies (Yagi, 2000; Shiroguchi and Toyoshima, 2001; Kikushima *et al.*, 2004), we propose that dynein activities are regulated by the concentration ratio of ATP and ADP, and that the OAD of *oda6E279K* somewhat differs from the wild-type OAD in this nucleotide-based motility regulation.

### The E279K mutation of IC2 is apparently affecting the β HC function

The phenotypes of various HC-deficient mutants in the background of the E279K mutation showed that this IC2 mutation lowers the motility in an α or γ HC-deficient mutant, but not in a mutant lacking the β HC motor domain (Fig. 6). Thus, this mutation might affect the OAD motor activity through the β HC. Interestingly, in the background of the IC2 mutation, *oda2-t*, a mutant that lacks the γ HC, showed lower motility than the mutant *oda1* lacking the entire OAD (Fig. 6). OAD αβ HCs with the mutant IC2 might somehow positively suppress the movement generated by IAD. As discussed below, IC2 may be interacting with an adjacent OAD aligned on the microtubule. Possibly, the mutant IC2 exerts some inhibitory effect on the β HC of adjacent OAD, as well as on the OAD to which it binds.

### Effect of the E279K mutation on the OAD motor activity

How does the E279K mutation of IC2 affect the OAD motor activity? One possibility is that the mutation may suppress the activity through a change in the structure of the OAD containing this IC2. Recent cryo-EM studies on the structure of OADs aligned on ciliary doublet microtubules revealed that the HC head domain is ~10 nm separated from the IC/LC complex, which is attached at the tail of an OAD molecule (Rao *et al.*, 2021; Walton *et al.*, 2021; Kubo *et al.*, 2021; Zimmermann *et al.*, 2023; Walton *et al.*, 2023). Despite such a large separation, we could imagine that a change in the tail structure might well affect the motor activity of OAD, because a mutation in the tail region has been shown to affect the motor activity in a cytoplasmic dynein (Ori-McKenney *et al.*, 2010). The E279K mutation could affect the structure of IC2 and the OAD tail, thereby resulting in a change in the motor activity of these HCs and the whole OAD.

**Fig. 7:**
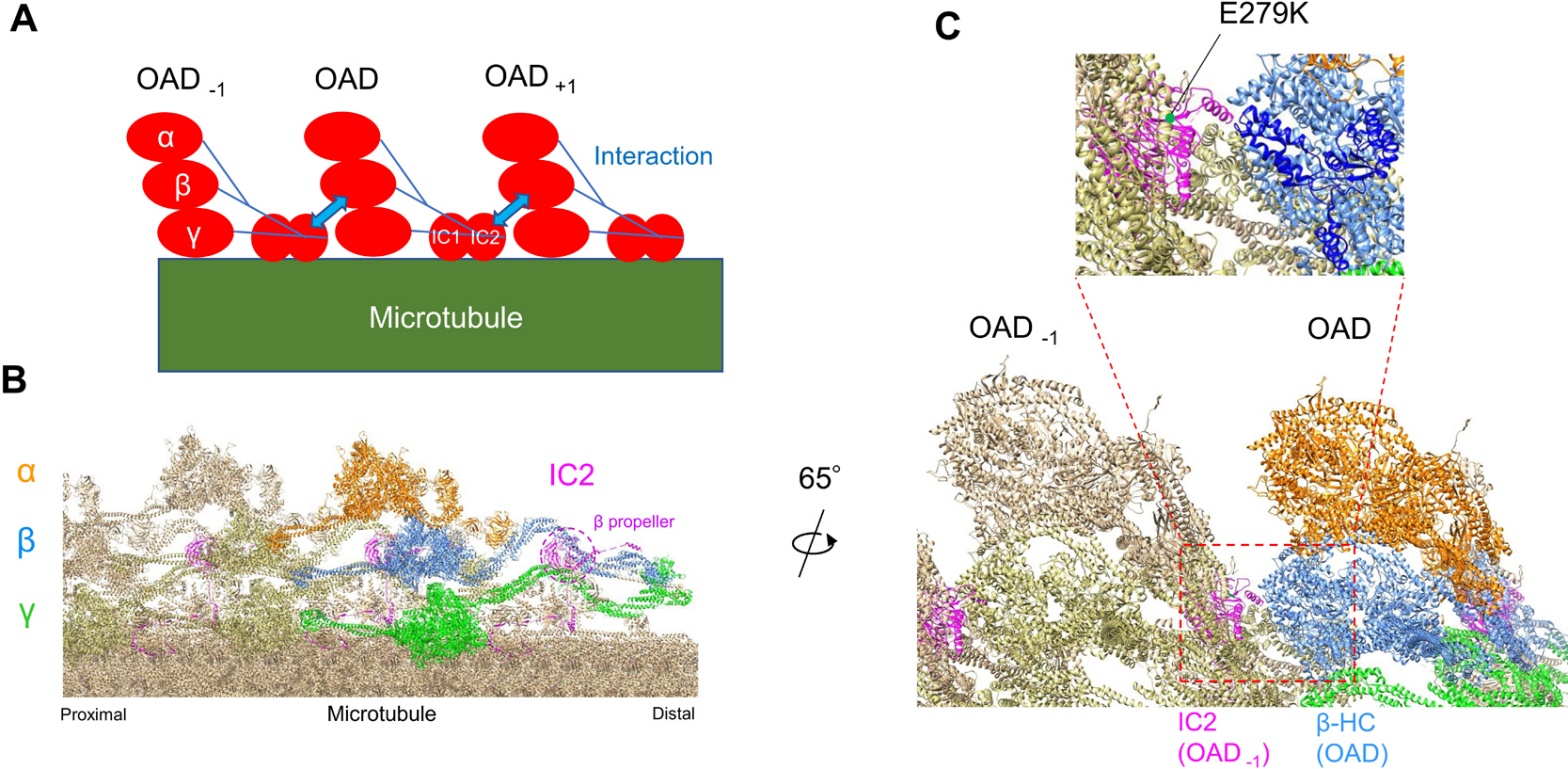
A model of IC2-OAD interaction A plausible model based on our findings and the structural data obtained by a previous study using cryo-EM (Walton *et al.*, 2023). (A) Cartoon of IC2-OAD interaction. OADs are regularly and tightly arranged on a doublet microtubule, enabling an IC2 subunit of an OAD molecule to interact with the motor domain of the β HC in the adjacent OAD. (B) The location of the IC2 based on Walton *et al.* (2023). A β-propeller structure in IC2 (shown in purple) attaches to the β-HC motor domain of an adjacent OAD. (C) The image on the left was rotated 65° and magnified. In the upper figure, the image around the IC2 in OAD_–__1_ and the motor domain of β HC in OAD was further magnified. The IC2 E279K mutation site (indicated) is located in the β-propeller structure. The AAA6 domain of β HC was shown in blue. These figures were drawn by modifying a cryo-EM image of *Chlamydomonas* outer doublet microtubule (PDB-8GLV). The UCSF chimera program (Pettersen *et al.*, 2004) was used.

At the same time, the OAD tail has been found to attach to the adjacent OAD head domains also (Walton *et al.*, 2021; Rao *et al.*, 2021, Kubo *et al.*, 2021). Specifically, the seven WD repeat domains of IC2, forming a β-propeller structure bound to the tail domains of both β and γ HCs, appear to also bind to the AAA6 domain in the head portion of the adjacent OAD β HC (Fig. 7C). Since the 279th glutamate residue of IC2 is likely located at the inner-side of the β-propeller and cannot directly interact with the AAA6 of the β HC (Fig. 7C), we surmise that the E279K mutation may well produce an allosteric structural change in the β-propeller portion to cause a strong impact. Previous studies suggested that adjacent OADs contained in microtubule bundles are likely to interact side-by-side or head-to-tail (Haimo and Fenton, 1984; Oda *et al.*, 2007; Aoyama and Kamiya, 2010). Inter-OAD interactions have also been detected in the axoneme by cryo-EM studies (Nicastro *et al.*, 2006; Ishikawa *et al.*, 2007; Rao *et al.*, 2021; Walton *et al.*, 2021; Kubo *et al.*, 2021; Zimmermann *et al.*, 2023).

The velocity of microtubule sliding has been shown to significantly increase when wild-type OADs are arranged tightly so that adjacent OADs can interact with each other; the speed increases up to 35 μm/sec, about twice the microtubule sliding velocity in disintegrating axonemes (Fig. 5B; Aoyama and Kamiya, 2010). In contrast, microtubule bundles formed with the OAD carrying the E279K IC2 displayed only a modest increase in the sliding velocity (Fig. 5B). The lack of a large increase in the sliding velocity in microtubule bundles formed with the mutant OAD suggests that the OAD with the E279K mutation of IC2 directly or indirectly weakens the OAD-OAD interaction on the microtubule *in vitro* (Fig. 7). This idea was supported by our EM observations. Namely, although the mutant OAD molecules were aligned between microtubules periodically like wild-type OAD, their alignment appeared less regular (Fig. 5D). To what extent the mutant and wild-type OADs differ in their ability to regularly align in microtubule bundles as well as in the axoneme must await further studies.

It must be noted that a decrease in the OAD-OAD interaction cannot readily explain the total behavior of the mutant’s axoneme. For example, microtubule sliding in disintegrating axonemes of the mutant occurred with a velocity of about 20 μm/sec, which was the same as that of the WT (Fig. 4A). We speculate that sliding with the wild-type velocity could occur in the mutant because an almost normal OAD-OAD interaction may take place in the axoneme, where OADs should be more precisely aligned than in an *in vitro* experiment. Alternatively, moderately fast microtubule sliding may possibly occur even without a strong OAD-OAD interaction in the axoneme, because IAD must also contribute to the sliding on the outer doublet. Another unexplained phenomenon is that the frequency of axonemal sliding disintegration is much lower in the mutant than in WT, while the frequency increases when the apparent dynein activity is enhanced by addition of ADP. We speculate that the mutant OAD has some deficiencies in nucleotide-induced conformational change, but it is puzzling why only the frequency of sliding and not the sliding velocity is reduced in the mutant.

To explore these unexplained observations, studies on the fine structures of OAD in the mutant axonemes, as well as in the microtubule bundles formed with the mutant OAD, will be necessary. Since it is unexpected that a single amino-acid substitution in a component located at the base of OAD causes such a gross change in the axonemal motility, we expect that further studies on this mutant will provide important clues to the mechanism of the cooperative activation of OAD, which we believe takes place during the oscillatory bending of cilia and flagella.

## Conflict of Interest

The authors declare that they have no conflict of interests with the content of this article.

## Abbreviations

PCD: primary ciliary diskinesia
IAD: inner-arm dynein
OAD: outer-arm dynein
HC: heavy chain
IC: intermediate chain
LC: light chain
AFLP: ampliﬁed-fragment-length polymorphism
SDS-PAGE: sodium dodecyl sulfate polyacrylamide gel electrophoresis
BCCP: biotin carboxyl carrier protein

## Supplementary Material

Movie S1 Lack of motility in the double mutant *ida4; 214*. The flagella of the double mutant *ida4; 214* are paralyzed.

Movie S2 Recovery of motility in the transformed *ida4; 214::IC2*. The double mutant *ida4; 214* displays swimming when transformed with the wild-type IC2 gene.
