# Supplementary material for: IC2 participates in the cooperative activation of outer arm dynein densely attached to microtubules
Source: Cell Struct Funct. 2023 Jul 27;48(2):175–85. doi: 10.1247/csf.23044 (PMC11496786; doi:10.1247/csf.23044)
Supplement: Supplementary file 3 — Supplementary Materials [file csf_48_23044_3.zip › 48_23044_3.docx]

Supplementary figures.


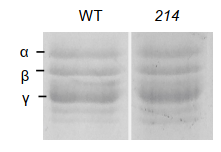


**Figure S1.** Dynein HC contents in the axonemes of WT and *214.*

Portions of 4% SDS-urea-PAGE patterns showing the high molecular weight bands. Gels were stained with Coomassie Brilliant Blue. Respective DHC bands are indicated.


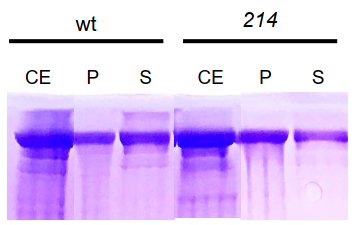


**Figure S2.** Microtubule co-pelleting assay for OADs from WT and *214*.

OAD was extracted from axonemes with 0.6 M NaCl. Crude dynein extract (CE) in HMDEK buffer (see Materials and Methods) was mixed for ten minutes with microtubules polymerized from purified porcine tubulin, and the mixture was pelleted by high-speed centrifugation. The resultant supernatants (S) and pellets (P) were analyzed by SDS-PAGE. The amounts of the WT OAD and mutant OAD bound to microtubules (the HC band intensities) in the pellet fraction are similar to each other, suggesting that microtubule binding affinity is not reduced in the mutant OAD. The concentration of dynein crude extract and tubulin is 0.8 mg/ml and 1.0 mg/ml, respectively.


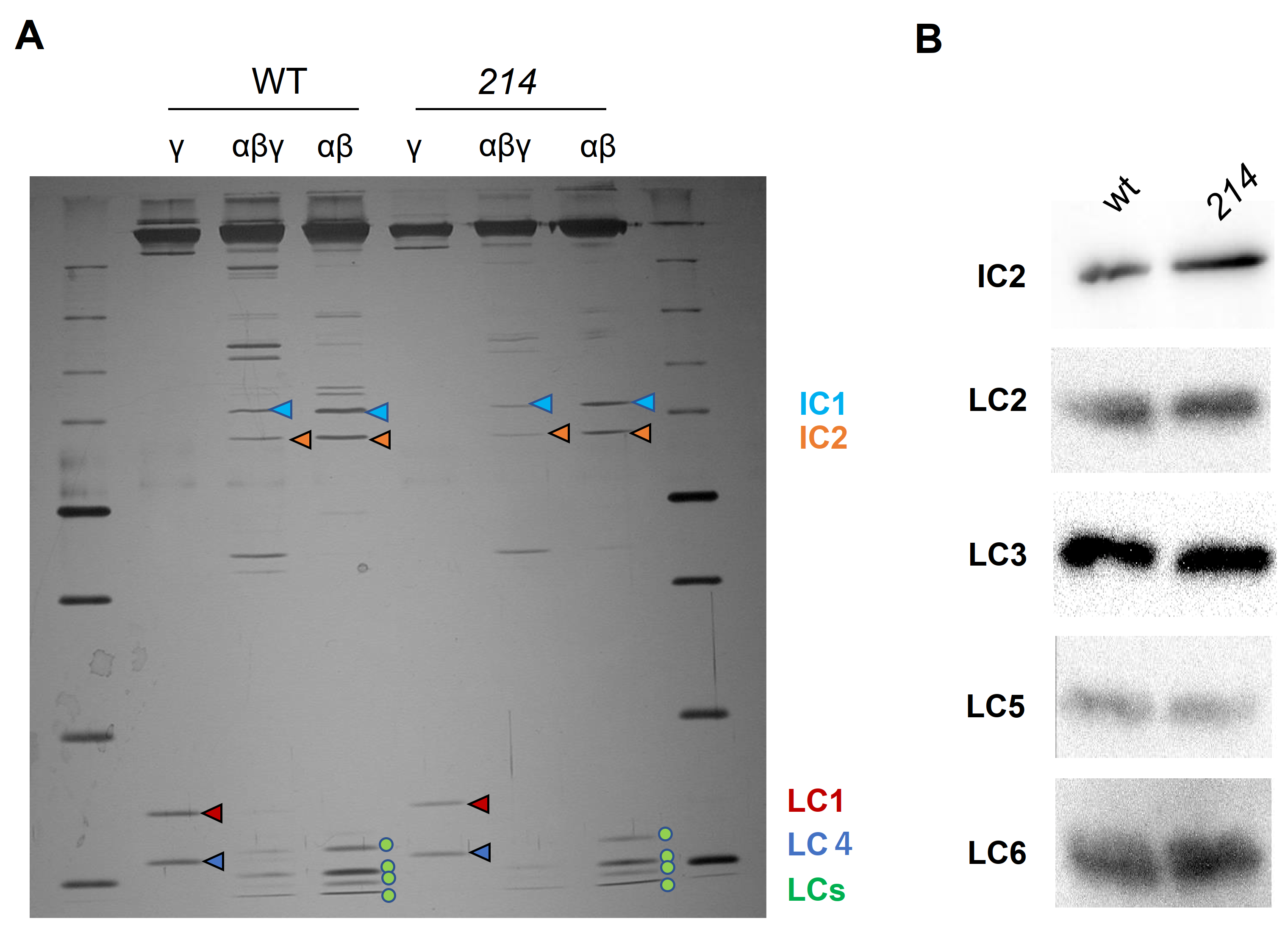


**Figure S3.** The contents of ICs and LCs in WT and *214* axonemes

(**A**) OAD species (γ, αβγ, αβ subcomplexes) were separated from high-salt axonemal extracts using ion exchange chromatography. OAD subunits were analyzed by SDS-PAGE on a 5-15% gradient gel. The band patterns and densities of ICs and LCs of *214* (*oda6 E279K*) appeared to be almost the same as those of WT. Arrowheads indicate the bands for IC1 (cyan), IC2 (orange), LC1 (brown), LC4 (blue), and circles indicate other LCs (green). (**B**) Immuno-blot analyses of IC2 and LC2, LC3, LC5, and LC6 in WT and *214* axonemes.


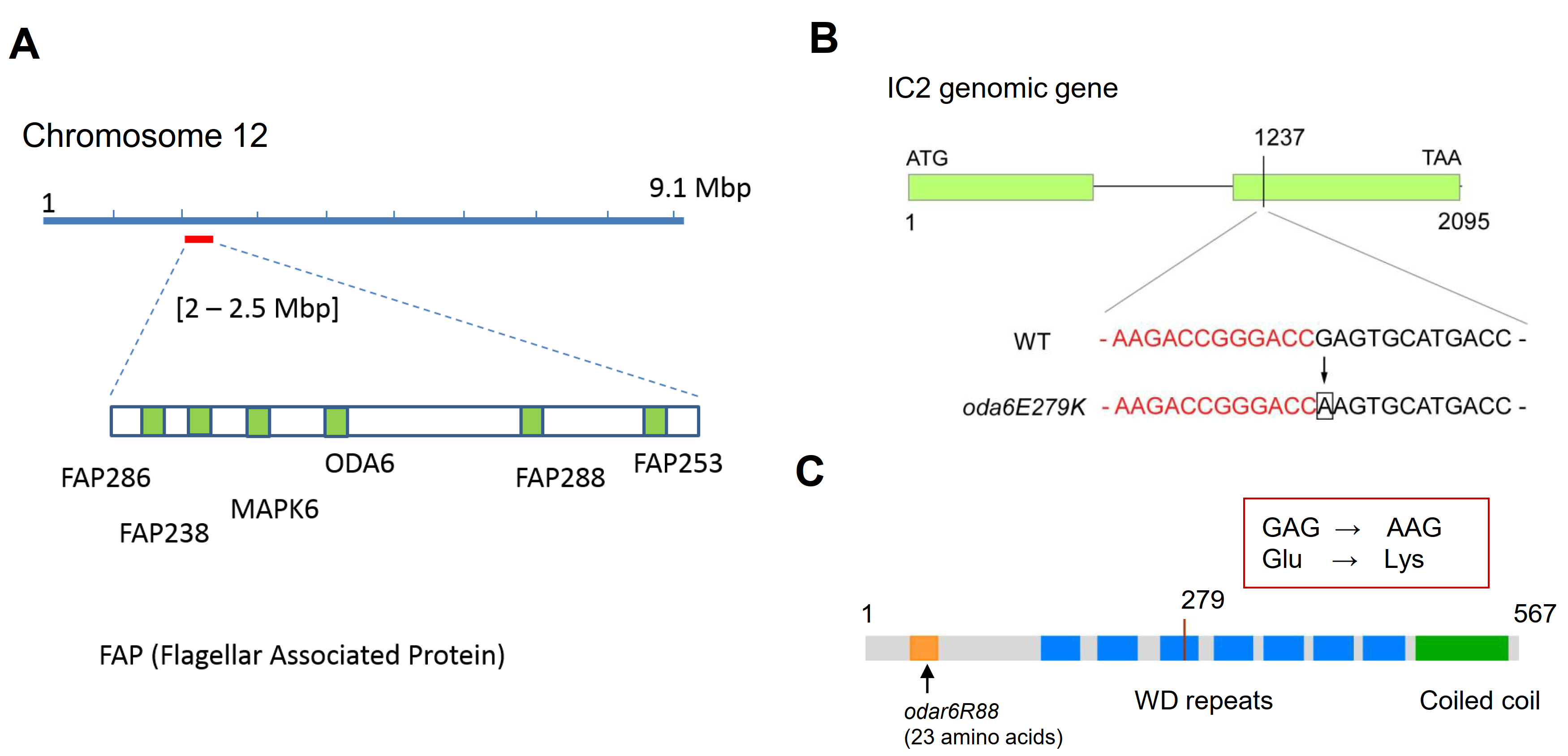


**Figure S4.** The *214* mutation is mapped to the gene encoding OAD intermediate chain 2.

(A) Amplified-fragment-length polymorphism (AFLP) mapping. The *214* mutation was mapped to a 2-2.5 Mbp region on chromosome 12. This region contains several genes of flagella associated proteins (FAPs) (Pazour et al., 2005), one of which is the *ODA6* gene encoding OAD intermediate chain 2 (IC2). (B) A G-to-A point mutation found in the *ODA6* gene. (C) The mutation causes a Glu to Lys change at the amino acid 279 located in the third WD domain.


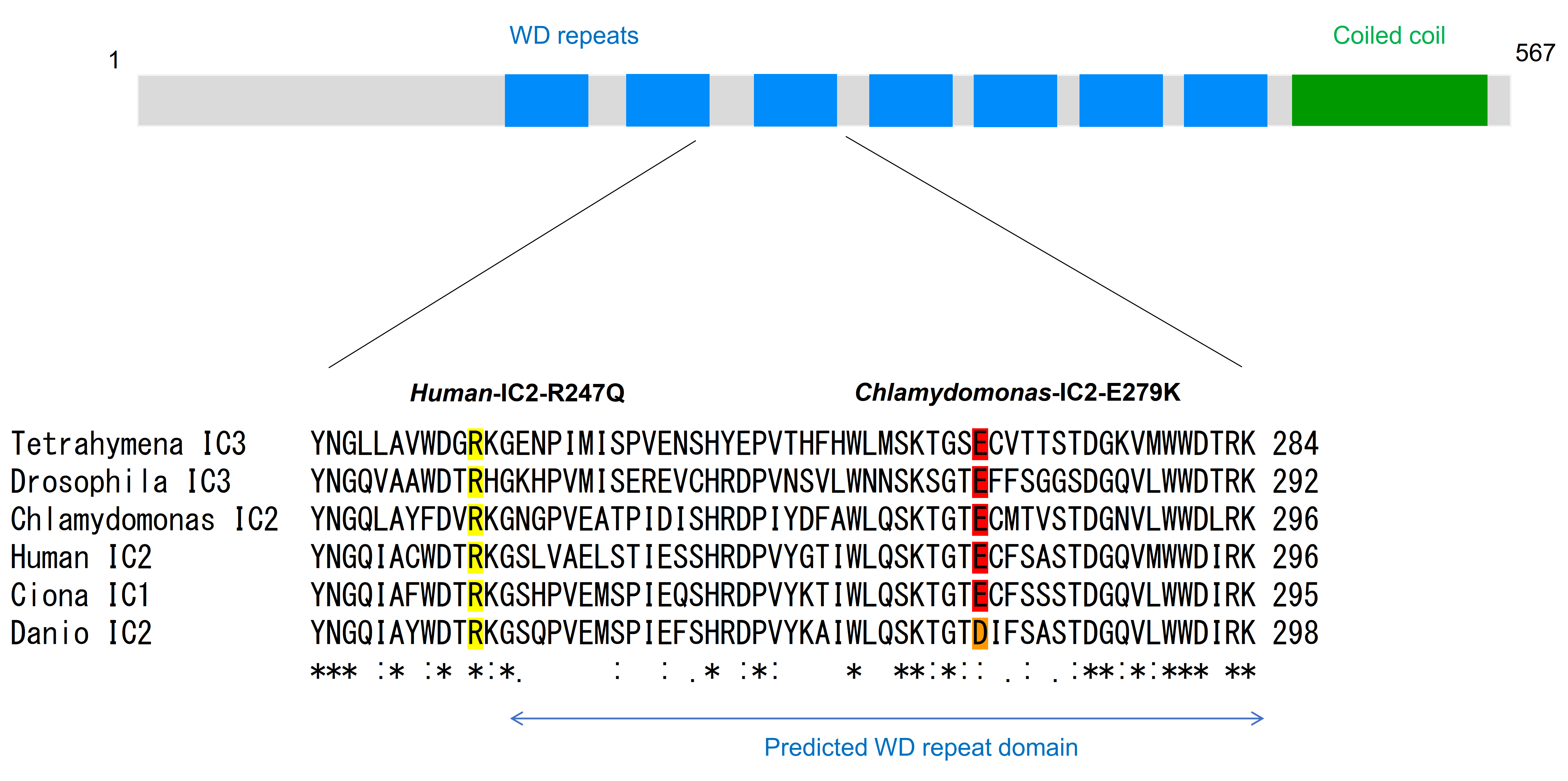


**Figure S5.** Comparison of OAD IC2 amino-acid sequences among various organisms.

The amino acid Glu at the 279th amino acid is conserved among many organisms except zebrafish, in which it is substituted by Asp. A mutation R247Q was previously found in the boundary region between the second and the third WD domains in human IC2. This mutation was found in a PCD patient who had a reduced number of OADs in trachea cilia (Al-Mutairi et al., 2022).


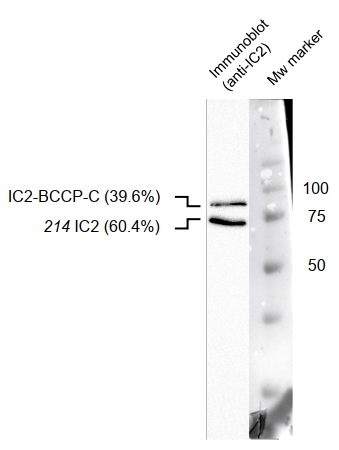


**Figure S6.** Contents of IC2 in the axoneme of *214::IC2-BCCP-C*.

Estimation of the relative contents of the mutant OAD (with an E279K IC2) and WT OAD in the axonemes of the transformant *ida4; 214(oda6E279K)::IC2*, using a strain transformed with a BCCP-tagged IC2 gene, *ida4; 214::IC2-BCCPC,* which expresses IC2 with biotin- carboxyl-carrier-protein (BCCP) sequence at the C-terminus (Oda et al., 2013). This transformant displayed almost the same vigorous motility as *ida4; 214::IC2*. Immuno-blot using IC2-specific antibody shows the band of IC2-BCCP-C and IC2, distinguished by their mobility difference. Their density ratio is approximately 0.4 (IC2-BCCP-C) : 0.6 (*214* IC2).

**Supplementary movies.**

**Movie S1.** Lack of motility in the double mutant *ida4; 214*.

The flagella of the double mutant *ida4; 214* are paralyzed.

**Movie S2.** Recovery of motility in the transformed *ida4; 214::IC2.*

The double mutant *ida4; 214* displays swimming when transformed with the wild-type IC2 gene.
